# Retraction: Shocked quartz at the Younger Dryas onset (12.8 ka) supports cosmic airbursts/impacts contributing to North American megafaunal extinctions and collapse of the Clovis technocomplex

**DOI:** 10.1371/journal.pone.0342620

**Published:** 2026-02-11

**Authors:** The PLOS One Editors

Following publication of this [1] article, concerns were raised regarding (a) the age model underpinning the results of this study; and (b) whether the conclusions are supported by the results presented.

The *PLOS One* Editors consulted with multiple experts in this field to investigate these concerns. They reviewed the article and found issues with (a) the underlying age model; and (b) the sampling strategy, which calls into question the validity of the reported conclusions.

Regarding (a), the underlying age model makes use of published radiocarbon dates; however, the authors have not included all of the available samples in their analysis, and at times it is not possible to determine what studies these samples originated from. Furthermore, the age range of the included dates range from centuries before and after the Younger Dryas onset, which calls into question the reliability of the attribution of the material in this study to the start of this period.

Regarding (b), the authors have not sampled the entire core section in their study. Therefore, it cannot be ruled out that there are spikes in their proxy record present at other depths of the sediment cores. This also precludes any analysis of whether the observed spikes exceed a background rate of this proxy, Furthermore, no age models have been reported, meaning it cannot be ruled out that the reported spike in cosmic airburst indicators resulted from changes in sedimentation rate as opposed to a true event.

In light of the above concerns that call into question the validity of the reported results, the *PLOS One* Editors retract this article. PLOS regrets that the issues were not identified prior to the article’s publication

JK, MLC, CM, GK, WW, SM, KL, and AW did not agree with the retraction. JJ, AM, VA, MY, TW, JR, VB, BD, JP, and RP either did not respond directly or could not be reached.
